# Long noncoding RNA *U90926* is crucial for herpes simplex virus type 1 proliferation in murine retinal photoreceptor cells

**DOI:** 10.1038/s41598-020-76450-2

**Published:** 2020-11-10

**Authors:** Shintaro Shirahama, Rena Onoguchi-Mizutani, Kentaro Kawata, Kenzui Taniue, Atsuko Miki, Akihisa Kato, Yasushi Kawaguchi, Rie Tanaka, Toshikatsu Kaburaki, Hidetoshi Kawashima, Yoshihiro Urade, Makoto Aihara, Nobuyoshi Akimitsu

**Affiliations:** 1 Department of Ophthalmology, Graduate School of Medicine, The University of Tokyo, Tokyo, Japan; 2 Isotope Science Centre, The University of Tokyo, Tokyo, Japan; 3 Division of Molecular Virology, Department of Microbiology and Immunology, The Institute of Medical Science, The University of Tokyo, Tokyo, Japan; 4 Department of Ophthalmology, Jichi Medical University Saitama Medical Centre, Saitama, Japan; 5 Department of Ophthalmology, Jichi Medical University, Tochigi, Japan; 6 Daiichi University of Pharmacy, Fukuoka, Japan

**Keywords:** Diseases, Molecular medicine

## Abstract

Long non-coding RNAs (lncRNAs) play vital roles in the pathogenesis of infectious diseases, but the role of lncRNAs in herpes simplex virus 1 (HSV-1) infection remains unknown. Using RNA sequencing analysis, we explored lncRNAs that were highly expressed in murine retinal photoreceptor cell-derived 661W cells infected with HSV-1. *U90926* RNA (522 nucleotides) was the most upregulated lncRNA detected post HSV-1 infection. The level of *U90926* RNA was continuously increased post HSV-1 infection, reaching a 100-fold increase at 24 h. Cellular fractionation showed that *U90926* RNA was located in the nucleus post HSV-1 infection. Downregulation of *U90926* expression by RNA interference markedly suppressed HSV-1 DNA replication (80% reduction at 12 h post infection) and HSV-1 proliferation (93% reduction at 12 h post infection) in 661W cells. The survival rates of *U90926*-knockdown cells were significantly increased compared to those of control cells (81% and 21%, respectively; *p* < 0.0001). Thus, lncRNA *U90926* is crucial for HSV-1 proliferation in retinal photoreceptor cells and consequently leads to host cell death by promoting HSV-1 proliferation.

## Introduction

Recent whole-transcriptome analyses using next-generation sequencing have clarified that a newly identified class of non-protein-coding transcripts, termed long noncoding RNAs (lncRNAs), are transcribed from a large proportion of the mammalian genome^1^. Increasing evidence suggests the involvement of lncRNAs in various biological processes, including regulation of transcription, splicing, and epigenetics. Furthermore, a large number of lncRNAs play important roles in cellular responses to stressful conditions^2,3^, including the response to infectious agents^4–8^. Consequently, lncRNAs could potentially be used as novel therapeutic targets for various diseases^9–11^.

Acute retinal necrosis (ARN) is a type of viral infectious retinitis caused by members of the herpes virus family, including herpes simplex virus type 1 (HSV-1) and varicella zoster virus^12^. Although antiviral agents, systemic corticosteroids, and anti-thrombotic drugs are used to treat ARN^13^, visual prognoses are very poor, mainly due to retinal photoreceptor cell death caused by viral infections^14^. Three classes of antiviral drugs (acyclic guanosine analogues, acyclic nucleotide analogues, and pyrophosphate analogues) have been approved for treating ARN, all of which target viral DNA polymerases^15^. However, long-term treatment with these antivirals leads to drug resistance, and thus, a novel antiviral drug for ARN is needed^15^.

The role of lncRNAs in HSV-1 infection remains unknown. Therefore, in this study, we used RNA sequencing analysis to investigate the lncRNAs that are regulated under HSV-1 infection in murine retinal photoreceptor cells (661W cells). Based on this analysis, we found that the lncRNA *U90926* was highly upregulated by HSV-1 infection, and therefore, we further focused on its role in the proliferation of HSV-1. We found that the knockdown of *U90926* markedly supressed HSV-1 proliferation and increased host cell survival in 661W cells, highlighting a potential new treatment target for ARN.

## Results

### Identification of *U90926* RNA as the most upregulated lncRNA in 661W cells infected with HSV-1

**Figure 1:**
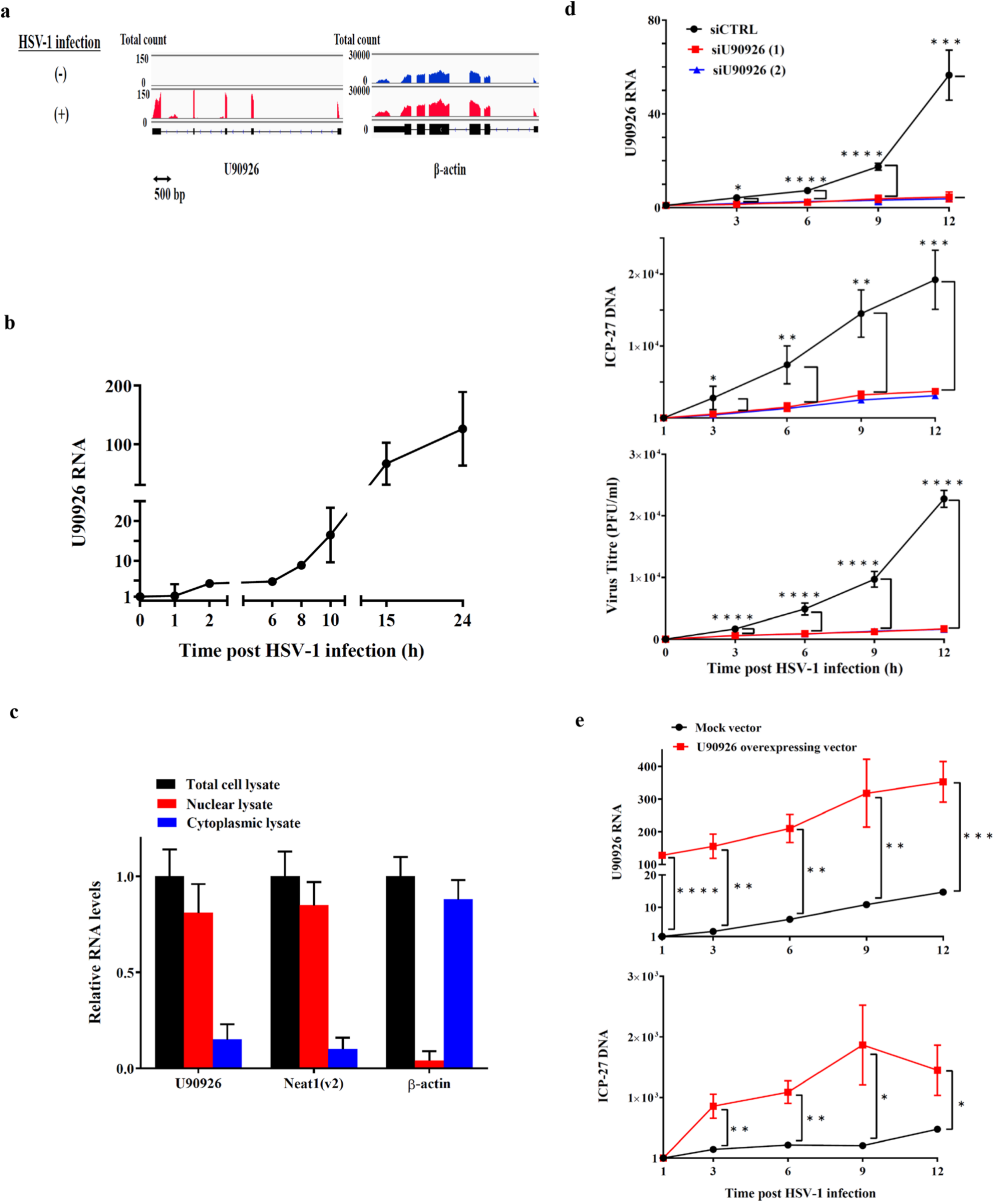

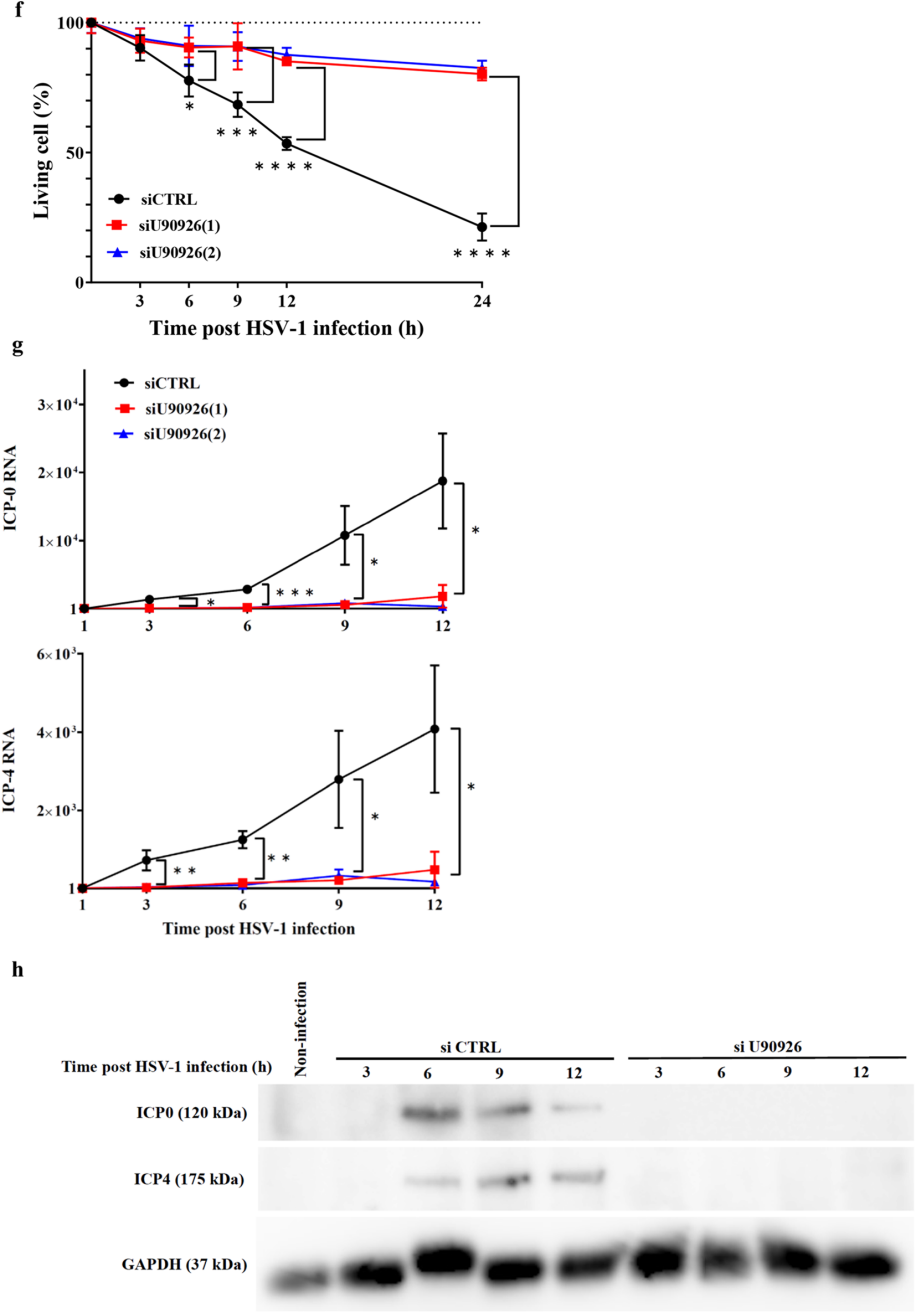
Induction kinetics and subcellular location of *U90926* RNA post HSV-1 infection, effect of *U90926* knockdown on HSV-1 DNA replication, proliferation, and genes expression. (**A**) RNA sequencing data of *U90926* and *β-actin* in non-infected (upper) and HSV-1-infected (lower) 661W cells, visualised using Integrative Genomics Viewer. (**B**) Time course of *U90926* RNA levels post HSV-1 infection (n = 3). (**C**) Relative RNA levels of *U90926*, *Neat1v2*, and *β-actin* in the whole cell (black), nucleus (red), and cytoplasm (blue) of 661W cells at 8 h post HSV-1 infection. *Neat1v2* and *β-actin* RNA served as the positive control for nuclear lysate and cytoplasmic lysate, respectively (n = 3). (**D**) Relative RNA levels of *U90926* (top), DNA levels of *ICP-27* (a HSV-1 gene) (middle), and HSV-1 titres (bottom) in control cells (black) or *U90926*-knockdown cells transfected with si*U90926*(1) (red) or s*iU90926*(2) (blue) at 3, 6, 9, 12 h post HSV-1 infection (n = 4). (**E**) Relative RNA levels of *U90926* (top), and DNA levels of *ICP27* (a HSV-1 gene) (bottom) in control or *U90926*-overexpressing cells at 3, 6, 9, and 12 h after HSV-1 infection (n = 3). (**F**) Survival rates in control cells or *U90926*-knockdown cells transfected with si*U90926*(1) (red) or si*U90926*(2) (blue) at 3, 6, 9, 12, and 24 h post HSV-1 infection (n = 4). (**G**) Relative *ICP0* (top) and *ICP4* (bottom) RNA levels in control (black) or *U90926*-knockdown cells transfected with si*U90926*(1) (red) or si*U90926*(2) (blue) at 3, 6, 9, and 12 h post HSV-1 infection (n = 3). (**H**) Immunoblot detection of ICP-0 (top) and ICP-4 (middle) proteins in control or *U90926*-knockdown cells at 3, 6, 9, and 12 h after HSV-1 infection. GAPDH (bottom) protein served as the loading control. All gels shown in this figure were cropped from different parts of the same jel. Values represent mean ± SD. *p < 0.05, **p < 0.01, ***p < 0.001, and ****p < 0.0001; Student’s *t*-test against control cells.

To identify HSV-1 infection-induced lncRNAs in 661W cells, ribosomal RNA-depleted RNAs from 661W cells with and without HSV-1 infection for 2 h were analysed by a massive sequencing approach. *U90926* RNA was identified as the most upregulated lncRNA in the infected cells (Supplementary Data 1). The annotated *U90926* transcript (NR_033483.1) in the National Centre for Biotechnology Information Reference Sequence database (https://www.ncbi.nlm.nih.gov/RefSeq/) was 522 nucleotides in length and comprised 5 exons (Fig. 1a). Analysis of the induction kinetics of *U90926* RNA post HSV-1 infection in 661W cells showed that the *U90926* RNA level gradually increased until 10 h post HSV-1 infection and then dramatically increased up to approximately 100-fold at 24 h post HSV-1 infection (Fig. 1b). Furthermore, cellular fractionation analysis revealed the nuclear localisation of *U90926* RNA in HSV-1-infected 661W cells (Fig. 1c). While non-infected samples showed almost no *U90926* transcripts (Fig. 1a).

Vascular endothelial cells are an important target in ARN^16^. Therefore, we infected retinal microvascular endothelial cells with HSV-1 and quantified *U90926* RNA and *ICP27* (a HSV-1 gene) DNA at 2, 4, 8, 12 h post HSV-1 infection. Consequently, *U90926* RNA was not detected before and after infection with HSV-1, although *ICP27* DNA were upregulated after HSV-1 infection (Supplementary Fig. 1). These results suggest that *U90926* RNA induction by HSV-1 infection is specific to retinal photoreceptor cells.

### Involvement of *U90926* RNA in HSV-1 proliferation

First, we evaluated the effect of *U90926* knockdown on HSV-1 replication and proliferation. We confirmed that the *U90926* RNA levels in *U90926*-knockdown cells, which were transfected with two different small interfering RNAs (siRNAs), were less than 20% those of the control cells at all time points evaluated post HSV-1 infection (Fig. 1d, top). HSV-1 DNA replication and proliferation in *U90926*-knockdown cells were then investigated by measuring the HSV-1 genomic DNA and HSV-1 titre, respectively. The *ICP-27* DNA level in *U90926*-knockdown cells was significantly lower than that in the control cells at 3 (*p* < 0.05), 6, 9 (*p* < 0.01), and 12 h (*p* < 0.001) post HSV-1 infection. At 12 h post HSV-1 infection, the HSV-1 DNA level in *U90926*-knockdown cells was decreased by about 80% compared with that in control cells (Fig. 1d, middle). In addition, the HSV-1 titre in *U90926*-knockdown cells was significantly (*p* < 0.0001) lower than that in control cells at 3, 6, 9, and 12 h post HSV-1 infection. At 12 h post HSV-1 infection, the HSV-1 titre in *U90926*-knockdown cells was decreased by about 93% compared with that in the control cells (Fig. 1d, bottom).

Next, we evaluated the effect of *U90926* overexpression on HSV-1 replication. We confirmed that *U90926* RNA levels in cells transfected with the *U90926* overexpressing vector were > 20-fold those in mock transfected (control) cells at 3, 6, 9, and 12 h post HSV-1 infection (Fig. 1e). Thereafter, we infected both cells with HSV-1, which significantly increased *ICP27* DNA levels in *U90926-*overexpressing cells compared with control cells at 3, 6 (*p* < 0.01), 9, and 12 h (*p* < 0.05) after HSV-1 infection (Fig. 1e). These results suggest that HSV-1 replication is accelerated by *U90926* RNA.

Further, we investigated whether *U90926* RNA was involved in the viability of HSV-1-infected cells. The viability of *U90926*-knockdown cells infected by HSV-1 was significantly increased compared to that of the control cells at 6 (*p* < 0.05), 9 (*p* < 0.001), 12, and 24 h (*p* < 0.0001) post HSV-1 infection. At 24 h post HSV-1 infection, the viability of *U90926*-knockdown cells was 80.2% [si*U90926*(1)] and 82.6% [si*U90926*(2)], whereas that of control cells was 21.3% (Fig. 1f).

### Role of *U90926* RNA in the expression of HSV-1 genes

HSV-1 replication is stimulated by the expression of HSV-1 early genes^17^. Furthermore, the expression of HSV-1 early genes depends on the expression of HSV-1 immediate early genes^18^. Therefore, we analysed the expression of several HSV-1 immediate early genes (*ICP0* and *ICP4*) in cells depleted of *U90926* RNA and found that *ICP0* and *ICP4* RNA levels in *U90926*-knockdown cells were significantly (*p* < 0.0001) lower than those in control cells at 3, 6, 9, and 12 h after HSV-1 infection. At 24 h after HSV-1 infection, *ICP0* and *ICP4* RNA levels were approximately 88% lower in control cells than in *U90926*-knockdown cells (Fig. 1g). Furthermore, in *U90926*-knockdown cells, the *ICP-0* and *ICP-4* proteins were not detected at 3, 6, 9, and 12 h after HSV-1 infection (Fig. 1h) (Supplementary Fig. 2).

### Identification of *U90926*-regulated host genes in HSV-1-infected 661W cells

To identify host genes regulated by *U90926* during an HSV-1 infection, we compared the expression of host genes between HSV-1-infected 661W cells in the presence or absence of *U90926* RNA. Finally, we identified upregulated differentially expressed genes upon HSV-1 infection and selected total 396 genes whose expression was completely suppressed in a *U90926*-dependent manner (Supplementary Data 2). Thereafter, we performed gene ontology enrichment analysis for the identified genes using PANTHER (https://www.pantherdb.org). For molecular function terms, gene ontology analysis revealed that differentially upregulated genes had significantly enriched immune-related functions, including ‘CXCR chemokine receptor binding’, ‘chemokine receptor binding’, ‘chemokine activity’, ’chemoattractant activity’, and ‘cytokine activity’ (Supplementary Fig. 3), suggesting that *U90926* RNA activates the host immune response. Accordingly, we propose a model wherein HSV-1 infection triggers *U90926* induction, thus upregulating HSV-1 immediate early genes and promoting HSV-1 DNA replication, ultimately supporting HSV-1 proliferation and activation of the host immune response.

## Discussion

We found that the lncRNA *U90926* was induced by HSV-1 infection in retinal photoreceptor cells and that it is a crucial factor for HSV-1 proliferation in these cells. A few reports have focused on the function of the lncRNA *U90926*. For example, *U90926* expression was demonstrated to be upregulated by Toll-like receptor stimulation in murine macrophages as a bifunctional lncRNA, acting as a positive regulator of interleukin-10 induction and as a negative regulator of CD80 and CD86^19^. In addition, *U90926* expression was reported to be downregulated during the differentiation of preadipocytes^20^.

*U90926* RNA is the first example of a lncRNA required for the proliferation of the viral DNA of HSV-1. Several lncRNAs are used to promote viral RNA proliferation in host cells, including *ACOD1* for the vesicular stomatitis virus^21^; *IPAN*, *PANN*, and *VIN* for type A influenza virus^22–24^; and *EGOT* for hepatitis C virus^25^.

Two different siRNAs (19 nucleotides) targeting *U90926* RNA efficiently suppressed the proliferation of HSV-1 in retinal photoreceptor cell-derived 661W cells and increased the survival rate of these cells. RNA interference therapy is considered useful for treating ophthalmological diseases, since the eye has a confined compartment and high accessibility, thus facilitating siRNA delivery^26^. However, siRNAs are generally unstable in the bloodstream and cannot efficiently cross the cell membrane; furthermore, they are immunogenic^27^. In eye, naked siRNA has been efficiently administrated by eye drop to the anterior segment or by intravitreal injection to the posterior segment of the eye^28^. For example, Fomivirsen (marketed as Vitravene) was the first Food and Drug Administration-approved antisense drug for cytomegalovirus retinitis with a 21-nucleotide sequence complementary to the immediate early region 2 of the cytomegalovirus mRNA, and administrated by intravitreal injection^29^. Considering these findings, lncRNA *U90926* is a potential novel therapeutic target for nucleic acid medicine to treat HSV-1 retinitis as it appears to be crucial for HSV-1 proliferation in retinal photoreceptor cells.

## Methods

### Cell culture

Both the murine retinal photoreceptor cell line 661W and Vero cells were obtained from Osaka Bioscience Institute. Both 661W and Vero cells were grown in Dulbecco’s modified Eagle’s medium (DMEM)/Ham’s F12 with l-glutamine and phenol red (661W) (Fujifilm Wako Pure Chemical Corporation) or high-glucose DMEM with l-glutamine, phenol red, HEPES, and sodium pyruvate (Vero) (Fujifilm Wako Pure Chemical Corporation), supplemented with 10% heat-inactivated foetal bovine serum (FBS) (Thermo Fisher Scientific) in a humidified incubator with 5% CO_2_. Retinal microvascular endothelial cells of BALB/c mice were obtained from Cell Biologics and were cultured in complete mouse endothelial cell medium/w Kit (Cell Biologics) supplemented with 10% heat inactivated FBS (Thermo Fisher Scientific) in a humidified incubator with 5% CO_2_.

### Plasmid constructs

To generate a *U90926* overexpressing vector, we purchased a pTwist CMV vector (Twist Bioscience) harbouring a part of the U90926 cDNA sequence (498 bp) between the NotI and BamHI site. This vector was used as a template to amplify full-length *U90926* cDNA using the forward 5ʹ-CACACACACACACACACACACACATATATATATATATGTT-3ʹ and reverse 5ʹ -TAATGTAAGCTTTTTATTGACACATCAGGTAGGGA-3ʹ primers. A linear vector was amplified with the forward 5ʹ -AAAAAGCTTACATTATCCGGACTCAGATCTCGAG-3ʹ and reverse primer, 5ʹ-GTGTGTGTGTGTGTGACCGGTAGCGCTAGCGG-3ʹ using pEGFP-C1 vector as the template. The whole *U90926* cDNA sequence was subsequently cloned into the linearized vector using In-Fusion HD Cloning Kit (Takara) in accordance with the manufacturer’s instructions.

### HSV-1 infection

HSV-1 (KOS strain) was propagated in monolayers of Vero cells. The 661W cells were seeded in culture plates 1 day before infection and were then challenged with HSV-1 at a multiplicity of infection of 5.

### RNA sequencing analysis

RNA-seq libraries for non-infection (n = 2) and infection samples (n = 2) (DRA008496) were constructed using the Ribo-Zero Gold rRNA Removal Kit (Epidemiology) (Illumina), according to the manufacturer’s instructions. Based on standard protocols, 100 bp paired-end-read RNA-seq data were generated using the Illumina HiSeq3000 sequencer. Further, we constructed RNA-seq libraries for non-infection samples (n = 2) (DRA008495), infection samples with control siRNA (n = 3), infection samples with si*U90926*(1) (n = 3), and infection samples with si*U90926*(2) (n = 3) (DRA010598) using the TruSeq Strand mRNA Sample Prep Kit (Illumina) in accordance with the manufacturer’s instructions. Using standard protocols, both 100 bp paired-end-read RNA-seq data for non-infection samples and 150 bp paired-end-read RNA-seq data for infection samples with siRNA were generated using the Illumina HiSeq3000 sequencer. The sequence data were aligned with the mouse genome sequence and annotation data (mm10) obtained from the Centre for Computational Biology at Johns Hopkins University (ftp://ftp.ccb.jhu.edu/pub/infphilo/hisat2/data/) using HISAT2 v2.1.0^30^, with default parameters. The aligned data were then input into StringTie v1.3.4d^31,32^ with default parameters to estimate the expression of individual transcripts as fragments per kilobase of transcript per million mapped reads (FPKM).

### Reverse transcription-quantitative real-time polymerase chain reaction (RT-qPCR)

Total RNA from HSV-1-infected 661W cells was isolated using the NucleoSpin RNA kit (Macherey–Nagel) and reverse-transcribed into cDNA using Prime Script RT master Mix (Takara). cDNA was amplified using the primer sets listed in Supplementary Table S1 using SYBR Premix Ex Taq II (Takara) in accordance with the manufacturer’s instructions. qPCR was then performed using a Thermal Cycler Dice Real Time System (Takara). *Gapdh* mRNA was used for transcript normalisation.

### Subcellular fractionation

Nuclear and cytoplasmic fractions were separated using a PARIS kit (Thermo Fisher Scientific) according to the manufacturer’s instructions. The RNAs in the nucleus and cytoplasm of 661W cells were extracted, and the expression levels of *U90926* in the nucleus and cytoplasm were examined by RT-qPCR. Neat1(v2) and β-actin were detected as fractionation indicators.

### Transfection of siRNA and *U90926* overexpressing vectors

All siRNAs were purchased from Thermo Fisher Scientific. The siRNA sequences were listed in Table S3. Both siRNAs and *U90926* overexpressing vectors were transfected into 661W cells by electroporation using a 4D-Nucleofector X Unit (Lonza) according to the manufacturer’s instructions. In brief, 661W cells were transfected with either siRNA duplexes (final concentration: 100 nM) mixed with SF solution (Lonza) or *U90926* overexpressing vectors (final concentration: 10 ng/μL) mixed with SF solution (Lonza), using the program EN-138.

### qPCR for HSV-1 DNA

Whole DNA was extracted from HSV-1-infected 661W cells using the NucleoSpin RNA kit with the NucleoSpin RNA/DNA buffer set (Macherey-Nagel). HSV-1 DNA was quantified by qPCR using specific primers for the *ICP27* gene of HSV-1, and the results were normalised against *Gapdh*^33^. The relative HSV-1 DNA amount was calculated based on a 1 h post HSV-1 infection sample.

### HSV-1 plaque assay

Serial dilutions of HSV-1-containing samples were applied to monolayers of Vero cells (12-well plate) and incubated at 37 °C for 1 h to allow virus attachment and entry into the cells. The supernatants were aspirated, and cells were washed twice with PBS(−) (Fujifilm Wako Pure Chemical Corporation) and incubated in 1% FBS-DMEM (high glucose) with l-glutamine, phenol red, and sodium pyruvate (Fujifilm Wako Pure Chemical Corporation) containing 0.2% γ-globulins (Merck) for 3 days at 37 °C with 5% CO_2_ to allow for plaque formation. The HSV-1 titre was calculated by plaque counting and expressed as plaque-forming units (PFU)/mL.

### Western blotting

Total protein extracts were prepared using cell lysis buffer (50 mmol/L Tris-HCl [pH 7.4], 1 mmol/L EDTA, 1% Triton X-100, 1% SDS, 5% Glycerol), and equal amounts of protein were separated on 4–15% Mini-PROTECAN TGX Precast Gels (Bio-Rad). The proteins were transferred onto a polyvinylidene fluoride membrane (Millipore) and blocked for 30 min at 25 °C with 3% BSA/Tris-buffered saline solution that contained 0.1% Triton X-100. Thereafter, the membrane was incubated with a 1:300 dilution of anti-ICP0 mouse monoclonal antibody (Abcam, ab6513), 1:5,000 dilution of anti-ICP4 mouse monoclonal antibody (American Type Culture Collection, hb-8183), and 1:5,000 dilution of anti-GAPDH mouse monoclonal antibody (Millipore, MAB374) for 1 h at 25 °C. All first antibodies were diluted with IMMUNO SHOT reagent 1 (Cosmobio). Thereafter, the membrane was incubated with a 1:5000 dilution of anti-mouse immunoglobulins goat polyclonal antibody conjugated with horseradish peroxidase (HRP) (Dako, P0447) for 1 h at 25 °C. The secondary antibody was diluted with IMMUNO SHOT reagent 2 (Cosmobio). Lastly, the proteins were visualized using Immobilon Forte Western HRP substrate (Merck) and chemiluminescence signal was detected with a Luminescent Image Analyzer LAS-4000 mini (Fujifilm).

### Measurement of 661W cell viability upon HSV-1 infection

The viability of 661W cells seeded in 96-well plates at 3, 6, 9, 12, and 24 h post HSV-1 infection was assayed using Cell Counting Kit-8 (Dojindo) according to the manufacturer’s instructions. We calculated cell viability using the following formula: [(absorbance values at a wavelength of 450 nm in 661W cells transfected with the indicated siRNA at 3, 6, 9, 12, and 24 h post HSV-1 infection) − (absorbance values at a wavelength of 450 nm in culture medium alone)]/[(absorbance values at a wavelength of 450 nm in non-infected 661W cells at each time point) − (absorbance values at a wavelength of 450 nm in the culture medium alone)]. Absorbance was measured using a Tecan Infinite F200 Microplate Reader (Tecan).

### Identification of upregulated differentially expressed genes completely suppressed in a *U90926*-dependent manner

Eleven RNA sequencing data were divided into four groups [A: non-infection without si control (n = 2), B: HSV-1 infection with si control (n = 3), C: HSV-1 infection with si*U90926*(1) (n = 3), D: HSV-1 infection with si*U90926*(2) (n = 3)]. In all groups, FPKM values were determined and upregulated differentially expressed genes completely suppressed in a *U90926*-dependent manner were selected on the basis of the mean FPKM value in each group. If the mean FPKM value in group A was > 0, those genes meeting all following criteria were selected; the mean FPKM value in group B was > 1, the value that [(mean FPKM value in group B)/(mean FPKM value in group A)] was > 5, the value that [(mean FPKM value in group C)/(mean FPKM value in group B)] was < 0.2, and the value that [(mean FPKM value in group D)/(mean FPKM value in group B)] was < 0.2. While, if the mean FPKM value in group A was 0, those genes meeting all following criteria were selected; mean FPKM value in group B was > 1, the value that [(mean FPKM value in group C)/(mean FPKM value in group B)] was < 0.2, and the value that [(mean FPKM value in group D)/(mean FPKM value in group B)] was < 0.2.

### Statistical analysis

Values are shown as the mean ± standard deviation. Comparison of continuous variables between two groups was performed using Student’s *t*-test. A *p* value less than 0.05 was considered significant. Student’s *t*-test was performed using GraphPad Prism software version 7 (GraphPad Software).

## Supplementary information

Supplementary information 1.

Supplementary information 2.

Supplementary information 3.

## Data Availability

The materials and datasets used and analysed during the current study can be obtained from the corresponding author on reasonable request.
