# Supplementary material for: Long noncoding RNA U90926 is crucial for herpes simplex virus type 1 proliferation in murine retinal photoreceptor cells
Source: Sci Rep. 2020 Nov 10;10:19406. doi: 10.1038/s41598-020-76450-2 (PMC7656448; doi:10.1038/s41598-020-76450-2)
Supplement: Supplementary file 1 — Supplementary information 1. [file 41598_2020_76450_MOESM1_ESM.docx]

**Supplementary Information**

**Long noncoding RNA *U90926* is crucial for herpes simplex virus type 1 proliferation in murine retinal photoreceptor cells**

Shintaro Shirahama, Rena Onoguchi-Mizutani, Kentaro Kawata, Kenzui Taniue, Atsuko Miki, Akihisa Kato, Yasushi Kawaguchi, Rie Tanaka, Toshikatsu Kaburaki, Hidetoshi Kawashima, Yoshihiro Urade, Makoto Aihara, Nobuyoshi Akimitsu

**Supplementary Figure Legends**

**Supplementary Fig. S1. Time course of HSV-1 DNA levels post HSV-1 infection in retinal microvascular endothelial cells.**

Time course of *ICP-27* (a HSV-1 gene) DNA levels post HSV-1 infection.

**Supplementary Fig. S2.** **Immunoblot detection of HSV-1 proteins in control or *U90926*-knockdown cells post HSV-1 infection.**

Immunoblot detection of ICP-0 (A) and ICP-4 (B) proteins in control or *U90926*-knockdown cells at 3, 6, 9, and 12 h after HSV-1 infection. GAPDH (C) protein served as the loading control. The same membrane was cut in the position of the black arrow. The following protein ladders were used. Protein ladder 1 : Blue Prestained Protein Standard, Broad Range (11-190 kDa) (New England Biolabs, P7706). Protein ladder 2 : PageRuler Prestained Protein Ladder, 10 to 180 kDa (Thermo Fisher Scientific, 26616).

**Supplementary Fig. S3. Gene ontology analysis of upregulated differentially expressed genes whose expression were completely suppressed in a *U90926*-dependent manner.**

Bar charts showed the top 10 gene ontology terms for molecular function as ranked by fold enrichment. All top 10 gene ontology terms meet a false discovery rate of less than 0.05.

**Supplementary Table S1.** **List of oligo siRNAs and DNAs used in this study.**

**Supplementary Data S1. List of the long non-coding RNA genes whose expression were upregulated more than two-fold post HSV-1 infection, identified by RNA sequencing analysis.**

**Supplementary Data S2. List of the upregulated differentially expressed genes whose expression was completely suppressed in a *U90926*-dependent manner.**

**Supplementary Fig. S1.**

**Supplementary Fig. S2.**

**A**

**B**

**C**

**Supplementary Fig. S3.**

| **Supplementary Table S1. List of oligo siRNAs and DNAs used in this study.** | | | | |
| --- | --- | --- | --- | --- |
|  | | | | |
| siRNAs | | Sense sequence (5′-3′) |  | Antisense sequence (5′-3′) |
| Silencer select-si control | | GUACCUGACUAGUCGCAGA |  | UCUGCGACUAGUCAGGUAC |
| Silencer select-si U90926 (1) | | CCACUGAGCAGAAGAACUA |  | UAGUUCUUCUGCUCAGUGG |
| Silencer select-si U90926 (2) | | UGCUCAUACUGAUAAAGAA |  | UUCUUUAUCAGUAUGACCA |
|  | | | | |
| Primer pairs for qPCR | | Forward primer (5′-3′) |  | Reverse primer (5′-3′) |
| U90926 |  | GTGATTCTGATGGCCCTTCT |  | ATCTTGCCAGGGAATCTTGA |
| Neat1v2 |  | CTTGCCACACCTTGTCTTGC |  | TAGCTGGTGCATCCTGTGTG |
| ICP-0 |  | ACCACCATGACGACGACTC |  | AGCCCCGTCTCGAACAGT |
| ICP-4 |  | GCAGCAGTACGCCCTGA |  | TTCTGGAGCCACCCCATG |
| ICP-27 |  | TCCGACAGCGATCTGGAC |  | TCCGACGAGGAACACTCC |
| β-Actin |  | GTACCCAGGCATTGCTGACA |  | CGCAGCTCAGTAACAGTCCG |
| GAPDH |  | GGTCCCAGCTTAGGTTCATCA |  | CCAATACGGCCAAATCCGTT |
